# Update on the Pathophysiology of Yellow Fever Intoxication

**DOI:** 10.4269/ajtmh.26-0276

**Published:** 2026-08-11

**Authors:** Amaro Nunes Duarte-Neto, Yeh-Li Ho, Esper G. Kallas, Adam L. Bailey

**Affiliations:** ^1^ Department of Pathology, Faculty of Medicine, University of São Paulo, São Paulo, Brazil;; ^2^ Department of Infectious Diseases and Tropical Medicine, School of Medicine, University of São Paulo, São Paulo, Brazil;; ^3^ Department of Pathology and Laboratory Medicine, School of Medicine and Public Health, University of Wisconsin-Madison, Madison, Wisconsin, USA

## Abstract

Yellow fever virus (YFV) remains a major global health threat, driven by climate change, vector expansion, and vaccine shortages. Despite centuries of observation, the pathophysiology of severe yellow fever (YF), specifically the highly lethal “intoxication phase," remains poorly understood. Recent clinical data from the 2016–2019 São Paulo epidemic and advances in animal models have shifted the understanding of YFV from a purely hepatocentric disease to a complex multiorgan syndrome. Emerging evidence indicates that YFV-induced endothelial damage and hypotension trigger profound intestinal ischemia and mucosal barrier breakdown, which allows gut bacteria to translocate into the portal system. Because the YFV-damaged liver cannot clear these pathogens, secondary polymicrobial sepsis ensues, likely contributing to the high mortality rates observed in severe YF. Our proposed gut-centered framework explains the distinct clinical presentation of YF intoxication. Recognizing secondary sepsis and vascular leakage highlights the urgent need for optimized fluid management, early dialysis, and targeted antimicrobial therapies to improve clinical outcomes.

## INTRODUCTION

**Figure 1.**
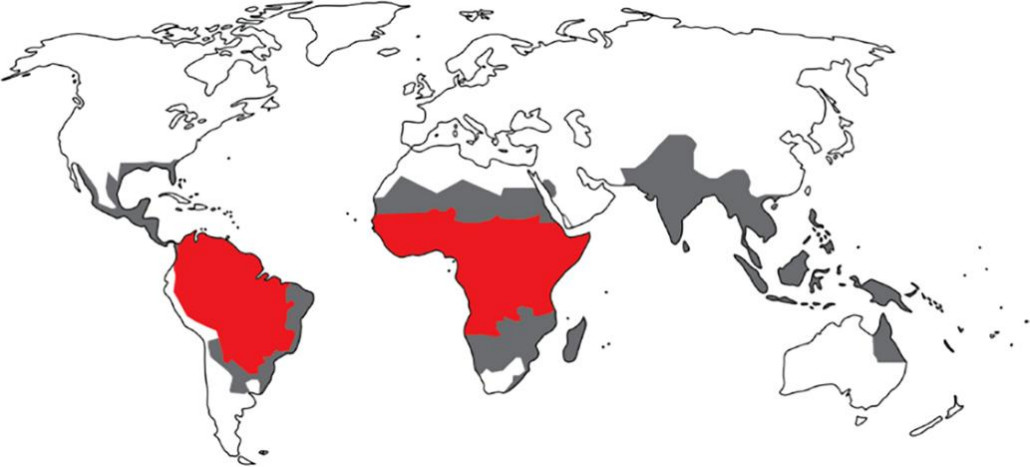
Geographic regions with endemic yellow fever virus (red) or *Aedes aegypti* (gray).

Yellow fever virus (YFV), the prototypical orthoflaviviruses from which the genus and family “*flavi*” (Latin for “yellow”) name is derived, is endemic in equatorial Africa and South America, where “sylvatic cycles” among nonhuman primates and forest-dwelling mosquitoes (e.g., *Hemagogus* and *Sabethes* mosquitoes) maintain a reservoir for zoonotic transmission to humans.^1^ Spillover into humans can facilitate a switch to the *Aedes* spp. mosquito vector that has a preference for feeding on humans, resulting in outbreaks that can threaten larger population centers in an “urban cycle” of transmission. The burden of yellow fever (YF) disease is challenging to measure, but modeling suggests that ∼130,000 severe cases and ∼78,000 deaths occur annually in Africa alone.^2^^,^^3^ Several factors (e.g., climate change, global warming, land use practices) are changing reservoir and vector habitats in ways that are expanding the frequency and endemic footprint of nearly all orthoflaviviruses, including YFV. This expansion includes larger and more frequent outbreaks in current endemic zones, as well as the emergence of YF in new and poorly prepared places.^3^ Although this may include occasional season-dependent outbreaks of YF in the southern United States and Europe, a greater concern is that spread to Asia could lead to the establishment of permanent YFV reservoirs in wild primates, putting large populations of unvaccinated people at risk.^4^^–^^7^ Currently, ∼2 billion people live in areas infested with *A. aegypti*, with human population growth and *A. aegypti* range expansion expected to increase this number dramatically in the coming decades (Figure 1). Although a highly effective YF vaccine is available, several factors complicate its implementation, including antiquated production technology resulting in vaccine shortages; a long list of vaccine contraindications and a well-established risk of severe complications that render many demographics ineligible; and the vast and rugged geography that hinders health care delivery in much of YFV’s area of endemicity.^8^ Hundreds of millions of people living in YF-endemic areas are currently in need of YF vaccination.^7^ Given the lack of YF-specific interventions post-infection, care for YF victims remains supportive and resource-intensive.

### Overview of YF disease course.

The course of YF has undergone several conceptual reorganizations since the mid-19th century. Initially, YF was described simply as “biphasic,” but with greater scrutiny, this delineation evolved to comprise several distinct phases or “periods.” Current conceptualization of the clinical course of YF describes the disease in four distinct phases/periods: incubation, infection, remission, and intoxication. During the “incubation period,” limited YFV undergoes initial replication within dermal dendritic cells proximal to the site of inoculation. The virus spreads to regional lymph nodes for further amplification, leading to a primary viremia that seeds the liver—the primary target tissue of YFV. Subsequently, YFV infects and kills hepatocytes, resulting in a secondary viremia. During this “period of infection,” individuals are acutely ill with nonspecific signs (hyperemia, fever) and symptoms (malaise, headache, myalgia, nausea, vomiting). This period is followed by a “period of remission” or “defervescence” during which most patients experience progressive clinical improvement leading to complete recovery within a few days. However, in ∼30% of cases, disease may “relapse” after 2 to 24 hours. This stage of the disease, known as the “intoxication phase,” is associated with high lethality and is characterized by a return of fever, worsening nausea, severe hepatitis (i.e., hepatic tenderness and jaundice), metabolic acidosis, multiorgan failure, and hemorrhagic manifestations such as “black vomit.”^9^^,^^10^

### Molecular pathogenesis of YF: A hepatocentric perspective.

The liver is the primary organ of YFV infection; as such, the hepatic pathology of YF from fatal cases is well described.^1^^,^^11^ Macroscopically, the YF liver is enlarged, congested, and steatotic.^12^^,^^13^ Microscopically, micro- and macro-steatotic degeneration is typical, with hepatocytes often displaying a “moruliform” appearance and the presence of eosinophilic inclusions known as Councilman-Rocha-Lima bodies.^11^^–^^14^ More recent studies employing electron microscopy and TUNEL staining have confirmed that Councilman bodies correspond to hepatocytes undergoing apoptosis.^15^^,^^16^ The distribution of these apoptotic cells, as well as that of viral antigens detected by immunohistochemistry, is mainly concentrated in the midzonal area of the hepatic lobule, suggesting a preferential viral tropism for hepatocytes within this region.^16^ Kupffer cells are also thought to be infected and killed,^17^ though this remains an understudied aspect of YFV infection. A mononuclear and neutrophilic inflammatory infiltrate is sometimes observed, and if present, is disproportionately mild in comparison with the extent of tissue damage. The mononuclear infiltrate, composed of lymphocytes and macrophages, tends to be more evident in the portal area and in zone 2, following the topography of hepatocellular lesions. Neutrophils are sometimes present within the portal tracts and perivenular interstitium, associated with venulitis and fibrinoid necrosis of the portal vein endothelium, as well as within the hepatic sinusoids. Edema in the portal tracts, sinusoidal congestion, and foci of hemorrhage, particularly in the midzonal region, are common. Preservation of the reticulin framework is a universal feature, with only sparse areas of mild collapse.^12^^,^^13^^,^^18^

### YF intoxication: Historical context.

The concept of the “intoxication”––which is central to the current understanding of YF disease––emerged gradually in the literature, first appearing in the work of physician Sinval Lins during the 1928–1929 YF outbreak in Rio de Janeiro, in which he stated, “Intoxication is everything; the infection is nothing, or almost nothing.”^19^^,^^20^ Intoxication was later discussed by Soper,^21^ and subsequently formalized by Kerr.^22^ The resemblance of YF intoxication to other hepatotoxic syndromes, including poisoning with carbon tetrachloride, phosphorus, arsphenamine, chromium, and chloroform, as well as hepatitis-inducing syndromes like yellow atrophy, cirrhosis, and eclampsia, underscored the idea that YF intoxication was a toxin-driven disease process. In the late 1920s to 1940s, therapeutic attempts to treat YF intoxication included the administration of glucose to counteract hypoglycemia and calcium salts to neutralize guanidine-like toxins; however, these interventions showed limited efficacy in both experimental models and clinical practice, and as summarized by Lins, “The disease cures itself of kills in spite of any and every treatment.” Thus, despite centuries of observation, the development of “intoxication” in YF––its predisposing factors and pathophysiology––remains poorly understood.

### YF in São Paulo: Revisiting intoxication with a modern clinical perspective.

Most cases of YF occur in remote settings with limited access to health care resources. An exception to this was the 2016–2019 YF epidemic in Brazil that reached the outskirts of São Paulo––a cosmopolitan city with large and sophisticated health care infrastructure––providing an unprecedented opportunity to treat, study, and improve the prognosis of intoxication-stage YF. Table 1 summarizes the major clinical and pathological studies that have resulted from this research so far. In this major outbreak, common clinical features of YFV infection at the time of hospitalization included fever, nausea, abdominal pain, hepatomegaly and hepatic tenderness, conjunctival injection, and Faget’s sign (fever with relative bradycardia).^23^ Many of the classic hallmarks of intoxication also manifested during hospitalization, including fulminant hepatitis with elevation of aspartate aminotransferase (AST) and alanine aminotransferase (ALT), coagulopathy, encephalopathy with increased seizure risk, arrhythmia, pronounced metabolic acidosis and profound lymphopenia.^24^^,^^31^ Neutrophilia was also surprisingly common.^23^ Gross hemorrhage was observed during the intoxication phase and predominantly involved the gastrointestinal tract––this was largely independent of prolonged prothrombin time or platelet count. Approximately half of YF-related deaths occurred within the first 72 hours of admission.^24^^,^^31^^,^^35^

**Table 1:** Clinical studies on yellow fever during the 2017–2019 Brazilian epidemics

First Author (year)	Place	Study Type/Period	Cases/Deaths (% lethality)	Demographics	Vaccine Status	Main Signs and Symptoms	Key Findings
Kallas (2019)^23^	São Paulo (FMUSP)	Observational cohort, January–May 2018	76/27 (36%)	89% male, median 42 years (32–54)	Not described	Fever, myalgia, headache, abdominal pain, bleeding	Older age, ↑neutrophils, ↑AST, ↑viral load linked to death. YFV clade SA I, subgenotype E.
Ho (2019)^24^	São Paulo (FMUSP)	Retrospective cohort, January–March 2018	79/53 (67%)	81% male, median 42 years (33–56)	86% unvaccinated	Fever, nausea, pain, bleeding, jaundice	High AST/ALT, metabolic acidosis, pancreatitis; 73% dialysis; CFR 67%; main causes: bleeding, acidosis, pancreatitis, MOF.
Freitas (2025)^25^	São Paulo (IIER)	Retrospective, January–March 2018	72/21 (29%)	80% male	13% vaccinated before onset	Fever, myalgia, nausea, vomiting	↑AST, ALT, creatinine independently linked to death.
Escosteguy (2019)^26^	Rio de Janeiro	Observational, March 2017–June 2018	52/21 (40%)	86% male, median 49 years	Not described	Fever, jaundice, vomiting, bleeding, renal failure	↑ALT >4000, ↑AST >6,000 associated with death.
Avelino-Silva (2023)^27^	São Paulo (FMUSP)	Observational cohort, January 2018–April 2019	84/30 (36%)	87% male, median 42 years (36–63)	Not described	Severe disease in 73%	↑YFV RNA correlated with death, ↑neutrophils, AST, INR, bilirubin, creatinine.
Arantes (2022)^28^	São Paulo (FMUSP)	Prospective cohort, Jan 2018–Apr 2019	95/59 (62%)	84% male, median 42 years (31–55)	Not described	AKI in 73%; dialysis 74%	Proteinuria and ↑Na+ excretion predicted AKI and death.
Casadio (2019)^29^	São Paulo (FMUSP)	Retrospective cohort	62 ICU cases	81% male, median 45 years (35–58)	1 patient with vaccine strain detected	Not specified	Lipase + factor V (cutoffs 147.5 U/L, 56.5%) predicted death (R²=0.78).
de Sousa (2024)^30^	São Paulo (FMUSP)	Observational cohort	57/17 (43%)	89% male, median 44 years	Not described	YF–severe: 39; YF–nonsevere: 18	↑NS1, syndecan-1, TEER correlated with severity, viral load, death.
de Ávila (2020)^31^	Minas Gerais	Longitudinal cohort, January–June 2018	114/54 (47%)	92% male, median 48 years (15–81)	21% vaccinated	Fever, jaundice, abdominal pain, diarrhea	Death linked to ↑INR, hepatic encephalopathy, APACHE II score.
Van de Weg (2024)^32^	São Paulo (FMUSP)	Case–control, January–May 2018	54 cases	Median 44–50 years	Not described	Severe YF	↑Ang-2, sENG, MMP-2, MMP-9 in nonsurvivors versus survivors.
Casadio (2020)^33^	São Paulo (FMUSP)	Observational cohort	133/59 (44%)	–	Not described	–	Late-onset relapsing hepatitis
Gonçalves (2024)^34^	São Paulo (FMUSP)	–	26/81 (33%)	100% male, avg. 42 years	Not described	Wide range	Immature neutrophilia associated with death

AKI = acute kidney injury; ALT = alanine aminotransferase; Ang-2 = angiopoietin-2; AST = aspartate aminotransferase; avg. = average; CFR = case fatality rate; clade SA I = South America I clade; FMUSP = Faculdade de Medicina da Universidade de São Paulo; ICU = intensive care unit; IIER = Instituto de Infectologia Emílio Ribas; INR = international normalized ratio; MMP-2 = matrix metalloproteinase-2; MMP-9 = matrix metalloproteinase-9; MOF = multiple organ dysfunction; NS1 = nonstructural protein 1; RNA = ribonucleic acid; sENG = soluble endoglin; TEER = transendothelial electrical resistance; YF = yellow fever; YFV = yellow fever virus.

In addition to the classic manifestations of severe YF at autopsy,^11^ striking alterations within the gastrointestinal tract were observed, extending along the gastrointestinal tract from the stomach to the sigmoid colon. These abnormalities included significant distension, a thickened and hemorrhagic bowel wall with serosal petechiae and hemorrhage in the lumen of the stomach, duodenum, small intestine and ascending colon. The pancreas also exhibited surprisingly extensive hemorrhagic necrosis, with some cases progressing to fatal necrohemorrhagic pancreatitis. The intra-abdominal fat tissue, including the retroperitoneal fat and epiploic appendages exhibited variable steatonecrosis with hemorrhages. Microscopic evaluation demonstrated marked vascular congestion and endothelial injury, characterized by cellular swelling, fibrinoid necrosis, and the presence of fibrin thrombi, interstitial edema, multifocal hemorrhages and widespread mucosal ischemic damage. Gram-negative bacteria were also detected in the portal venous system and hepatic lobules, accompanied by a mixed inflammatory infiltrate.^12^^,^^13^^,^^36^

In post hoc analysis of the clinical data from this epidemic, several clinical and laboratory findings were independently associated with fatal outcomes, including advanced age, neutrophilia, elevated creatinine, elevated AST > ALT, high viral load (as measured by real-time quantitative polymerase chain reaction, reduced coagulation factor V, low fibrinogen, elevated D-dimer, metabolic acidosis, and increased lipase.^23^^,^^34^^,^^35^

Additional studies have examined the unusual abundance of neutrophils in YF intoxication. Gene expression analysis suggests that these neutrophils are highly immature and unlikely to have significant effector function.^34^ Progress has also been made toward identifying the stimulus for neutrophil mobilization. Several markers of bacteremia (e.g., 16s rDNA) and host responses to bacteremia (e.g., lipopolysaccharide [LPS] binding protein [LBP], and soluble [s]CD14) were significantly elevated in YF patients relative to controls. Increased levels of intestinal fatty acid binding protein (I-FABP)––a measure of intestinal damage––were also identified at the time of admission, suggestive of a gastrointestinal source.

### Leveraging animal models to understand YF intoxication.

Experimental YFV infection of macaque monkeys (particularly rhesus macaques, *Macaca mulatta*) leads to severe disease that recapitulates many of the hallmarks of intoxication-stage YF in humans, making the macaque model the longstanding gold-standard animal model of YF.^37^^,^^38^ However, modern-day cost and biosafety considerations have significantly limited the tractability of the macaque model for understanding intoxication.^39^ In contrast to primates, mice are naturally resistant to YFV infection.^40^ Although immunocompromised mice are susceptible, the pattern of infection and disease in these animals––referred to as “neurotropic” to contrast it with the “viscerotropic” pattern of infection observed in humans and rhesus macaques––limits the utility of this model for studying YF intoxication. Recently, mice engrafted with human hepatocytes have been used to study various aspects of YFV infection; however, this system has many limitations including distorted hepatic architecture, immature hepatocyte differentiation, dual neuro- and viscerotropism, and substantial defects in both innate and adaptive immunity.^41^^–^^44^ In the early 2000s, two “hamster-adapted” YFV strains were developed via in vivo serial passage, resulting in viruses that cause hallmarks of viscerotropism/intoxication (hepatitis, coagulopathy, lethality) in wild-type hamsters.^45^^,^^46^ YFV-infected hamsters also develop neutrophilia and bacteremia late in the course of disease, providing a system for modeling upstream events in the development of intoxication.^36^ So far, studies in the hamster model have shown that damage to the gastrointestinal tract (e.g., erosion of intestinal villi) provides a portal of entry for gut bacteria, which were found in the liver and systemic circulation later in the disease process. An important finding was that intestinal damage in this system was not due to YFV infection of cells in the gut; rather, histological changes pointed to early and profound ischemic injury (i.e., lack of blood supply) with hemorrhages in the lamina propria, submucosa, and muscular and subserosa intestinal layers.^36^

### A gut-centered framework for understanding YF intoxication.

**Figure 2.**
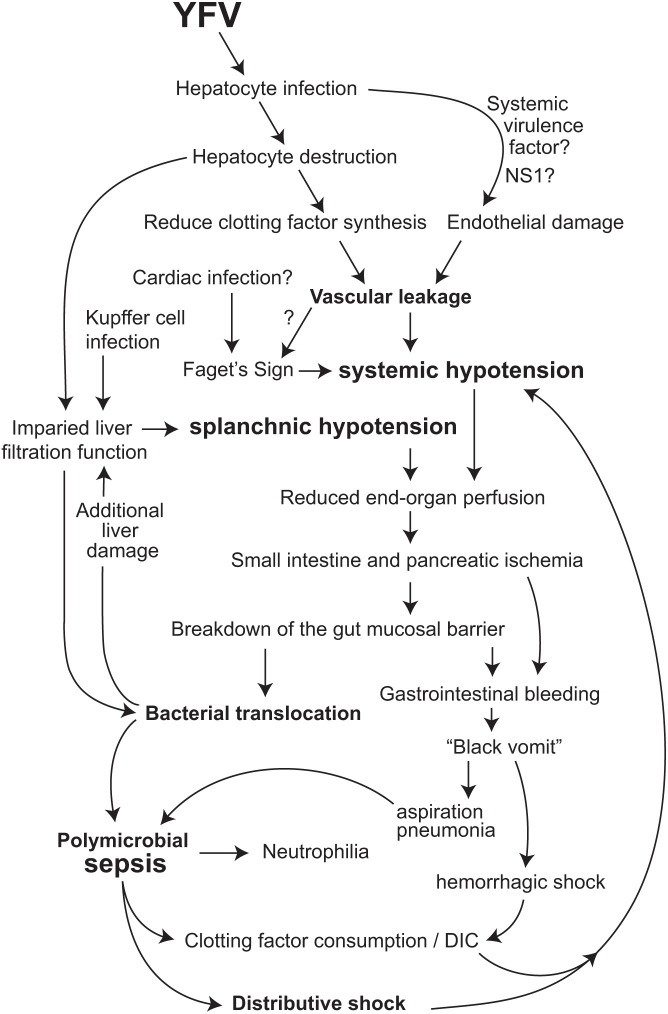
Updated model of the pathophysiologic cascade in yellow fever intoxication.

Multiple lines of evidence now implicate the gastrointestinal tract as a major player in YF intoxication.^12^^,^^13^^,^^36^ The pathophysiology that culminates in gastrointestinal injury remains to be fully elucidated and may be multifactorial. However, it is likely that endothelial damage is playing a significant role.^30^ Additionally, altered splanchnic circulation due to hepatic congestion may be involved. Regardless of the cause, gastrointestinal ischemic injury appears to precipitate a combination of intestinal hemorrhage and mucosal barrier breakdown. Luminal bacteria then enter this breach and track through the portal system to the liver. Typically, these bacteria would be phagocytosed by the highly active and abundant macrophages of the liver, Kupffer cells. However, damage to the hepatic architecture, as well as Kupffer cell infection and dysfunction,^17^ likely prevents the YFV-ravaged liver from performing this critical filtration function. Consequently, bacteria spill into systemic circulation causing a polymicrobial bacteremia, resulting in sepsis in a host already ravaged by YFV infection, likely contributing to the lethality of this condition. This mechanism likely explains why peripheral blood neutrophilia is a poor prognostic factor in critically ill patients (Figure 2).^23^^,^^34^^,^^36^

Secondary sepsis has rarely been studied in the context of YF but is relatively common when looked for.^31^ Multiple factors have likely contributed to the under recognition of secondary sepsis in YF, such as the rapid evolution of severe YFV and its occurrence in mostly remote settings with limited health care infrastructure. Many of the signs and symptoms of secondary sepsis also overlap with the signs and symptoms of YF, making the clinical diagnosis of sepsis on top of YF challenging. Finally, lack of systematic organ sampling in YF autopsies, possibly due to the focus on hepatopathology, may have historically limited the detection of sepsis-related findings.

### Upstream determinants of intestinal ischemia.

This new conceptualization of YF intoxication highlights several important knowledge gaps; in particular the upstream factors that contribute to intestinal and pancreatic ischemia. The small intestines are highly susceptible to ischemic injury, and it seems that hypotension, either systemically or locally within the splanchnic vasculature, may play a role in the gastrointestinal damage that precipitates severe YF intoxication in fatal cases. Multiple interrelated processes (summarized below) likely synergize to cause the unique blend of hypotension that is observed in YF, and establishing their relative contributions to the overall clinical picture will inform YF management.

First, it is likely that endothelial damage caused by the soluble viral protein NS1 plays a role in the development of vascular leakage. Although research on YFV-NS1 is relatively lacking, NS1 from multiple related orthoflaviviruses like dengue virus (DENV) have been shown to disrupt the endothelial glycocalyx later, resulting in endothelial injury and vascular leakage.^30^^,^^31^^,^^47^^–^^49^ In DENV with severe features, NS1-mediated endothelial damage also contributes to peripheral hypoperfusion, mucosal ischemia, bacterial translocation, and progression to shock.^50^ Growing evidence indicates that YFV-NS1 also mediates vascular leakage through a related mechanism; however, in YF, this process is likely further exacerbated by reductions in circulating clotting factors that are brought on by YFV infection/destruction of hepatocytes (the cell type responsible for clotting factor synthesis). Although vascular leakage at any anatomical location could contribute to systemic hypotension, some evidence also suggests that NS1 orthologs from different orthoflaviviruses may exhibit tissue-specific endothelial toxicity^51^; thus, it remains possible that local NS1-mediated injury to the splanchnic or mesenteric vasculature may play a role in the development of intestinal ischemia.

Second, the poorly understood febrile bradycardia of YF, known as “Faget’s sign,” may exacerbate intestinal ischemia via reduction in cardiac output. Recent examination of the heart tissue from autopsies of YF intoxication victims revealed fibrinoid necrosis of endothelial cells, interstitial edema, hemorrhage, and a mild inflammatory infiltrate composed predominantly of macrophages, with YFV antigen detected within endothelial and infiltrating leukocytes cells.^52^ Cardiac tissue also showed significant elevations in pro-inflammatory cytokines. Precisely how these changes ultimately translate into electrical conduction abnormalities in the heart remains to be determined.

Other well-established mechanisms of hypotension may also contribute to intestinal ischemia in YF, such as systemic vasodilation (i.e., “distributive shock”) caused by massive cytokine and inflammatory responses to viral and/or bacterial infection.^52^

### The lungs: Another source of sepsis in severe YF?

Secondary pneumonia is mentioned in some historical YF outbreaks, and respiratory tract infections were surprisingly common in the fatal cases from São Paulo: among 73 fatal cases, secondary pneumonia was identified in 84%, often associated with bronchoaspiration (60%) and micro-abscess formation. YF-associated pneumonias exhibit polymicrobial features, involving Gram-positive and Gram-negative bacteria as well as fungi such as *Candida* and hyalohyphomycetes.^12^^,^^13^ It is interesting to note that YFV antigens are commonly detected in the lungs—localized within capillary endothelial and inflammatory cells— and possibly indicative of direct viral injury to the pulmonary parenchyma, which may contribute to alveolar edema and hemorrhage. Exudative diffuse alveolar damage and microscopic thrombi are also common, both potentially exacerbating hypoxemia and coagulopathy.^53^ Collectively, these findings support the existence of a pathophysiological axis linking the gastrointestinal tract, liver, and cardiopulmonary system, which may explain the distinct “intoxication phase” of YF and distinguish it from classical fulminant viral hepatitis, which typically lacks direct extrahepatic injury or virus-induced endothelial damage during the acute phase.

### Recommendations for managing intoxication.

**Figure 3.**
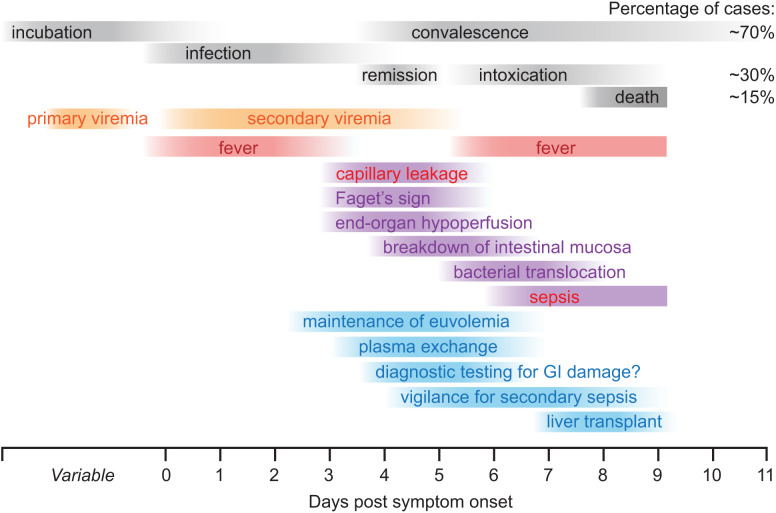
Proposed pathophysiology underpinning the “periods” of yellow fever (YF) disease progression. The classic periods/phases of YF disease over time are shown in gray, with the viremia and fever shown in orange and red, respectively. New insights and proposed mechanistic drivers of the “intoxication” phase are shown in purple, with potential countermeasures/interventions shown in blue.

Managing severe YF continues to be a major challenge for physicians, and a comprehensive guide to the care and management of YF patients can be found in the *Clinical Management Guide for the Severely Ill Patient with Yellow Fever Bulletin* published by the Pan American Health Organization (PAHO).^54^ An exciting development is that recent experiences have led to many innovations that are starting to provide a clearer picture of lifesaving management strategies. These include early dialysis and correction of metabolic acidosis, prophylactic anticonvulsant therapy, and plasma exchange to remove circulating cytokines and toxic metabolites and replenish coagulation factors. Gastrointestinal-protective measures such as proton pump inhibitors may also be useful. Given the emerging role for intestinal ischemia in the development of intoxication, further improvements may also involve closer attention to the careful maintenance of euvolemia (Figure 3).

**Figure 4.**
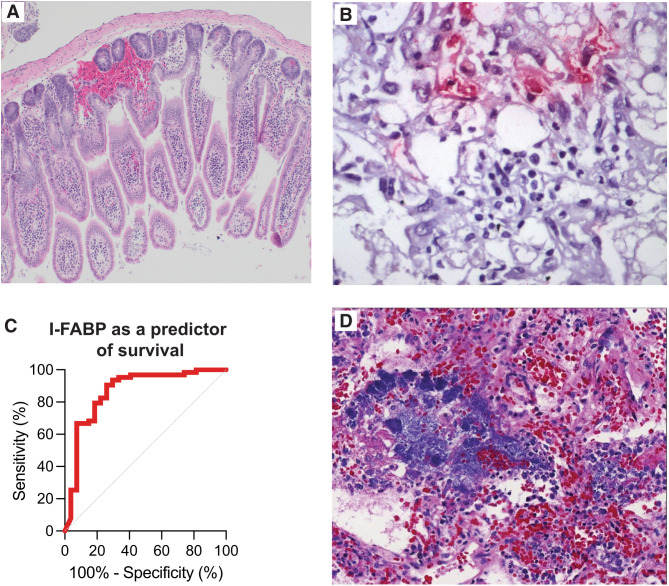
Historically overlooked features of yellow fever (YF) intoxication. (**A**) Submucosal hemorrhage in the small intestine of a yellow fever virus (YFV)-infected hamster. Abnormal swelling of intestinal villi can also be seen. (**B**) Lipopolysaccharide-positive material in the mid-zonal sinusoids, associated with lobular inflammatory reaction in the liver of a YF victim. (**C**) Receiver operator characteristic (ROC) curve of intestinal fatty acid binding protein blood concentrations, taken at the time of hospital admission, as a predictor of survival. (**D**) Polymicrobial pneumonia and alveolar hemorrhage in a YF victim.

Predicting the YF patients that will progress to intoxication will also guide medical management and allocation of medical resources. In DENV with severe features, which also has a component of bacterial translocation, radiological findings such as bowel-loop edema, ascites, and gallbladder wall thickening are early indicators of vascular leakage and predictive of increased mortality.^55^^–^^57^ Thus, the value of these noninvasive measures for determining the severity of vascular leak in YF seems worthy of further exploration. Blood tests will also play an important role, with closer attention to neutrophil counts along with the usual focus on liver function tests. Although a test for blood levels of I-FABP is not available clinically, preliminary data indicates that I-FABP levels are highly predictive of mortality, with a 90% sensitivity and 74% specificity at the Youden index maximum (Figure 4).^36^

Given that secondary sepsis is likely playing a causal role in YF lethality, efforts to understand the microbiology of this condition and optimize antimicrobial therapy are urgently needed. Currently, the WHO and the PAHO recommend treating YF-associated bacterial infections with antibiotics, if they occur.^54^^,^^58^ Although prophylactic antibiotics may seem attractive for the prevention of YF-associated bacteremia, this approach may have additional undesired consequences, such as predisposition to invasive fungal infection.^53^ Thus, future studies are needed to understand whether prophylactic antibiotics for the prevention of YF-associated bacterial infection is of clinical benefit.

Although much work remains to be done toward improving YF outcomes, a renewed interest in understanding YF pathophysiology brings hope for the treatment of this historically intractable disease.
